# The efficacy and safety of denosumab, risedronate, alendronate and teriparatide to treat male osteoporosis: a systematic review and bayesian network meta-analysis

**DOI:** 10.3389/fendo.2025.1579101

**Published:** 2025-06-19

**Authors:** Shu Jun Chai, Tao Yu, Guo Rui Wang, Fu Lu Sun, Hao Xue, Zun Zhang, Bo Ran

**Affiliations:** ^1^ The Third Affiliated Hospital of Inner Mongolia Medical University, Inner Mongolia, China; ^2^ The Second Hospital of Jilin University, Changchun, Hebei, China

**Keywords:** systematic review, denosumab, risedronate, alendronate, teriparatide, network meta-analysis

## Abstract

**Background:**

Male osteoporosis treatment lacks robust comparisons of efficacy and safety among key medications. This network meta-analysis (NMA) aimed to systematically evaluate alendronate (ALE), risedronate (RIS), teriparatide (TER), and denosumab (DEN) in male patients, addressing this critical evidence gap.

**Methods:**

Following PRISMA 2020 guidelines, we conducted a systematic review and NMA. Databases were searched for randomized controlled trials (RCTs) comparing these drugs in males with osteoporosis (PICOS criteria). Pairwise meta-analysis (Stata 18.0) assessed effect sizes, while NMA (R 4.3.1, gemtc and BUGSnet packages) ranked treatments for BMD changes (lumbar spine, femoral neck, total hip) and safety outcomes (adverse and serious adverse events).

**Results:**

From 2729 screened records, 12 studies were included. TER ranked highest for lumbar spine BMD improvement and overall safety (lowest adverse events). ALE showed superior femoral neck and total hip BMD gains but higher adverse event risks vs. TER. DEN improved BMD at all sites but had the poorest safety profile (highest adverse events). RIS was safest (lowest serious adverse events) but least effective for BMD enhancement.

**Conclusions:**

Teriparatide is the optimal choice for improving lumbar spine BMD and overall safety, while alendronate shows significant efficacy in enhancing femoral neck and hip BMD, although its safety profile is less favorable. Thus, alendronate may be more suitable for patients needing bone density improvement at these sites. Treatment choices should weigh site-specific needs against risk tolerance.

**Systematic review registration:**

https://www.crd.york.ac.uk/prospero, identifier CRD42024599021.

## 1 Introduction

Osteoporosis is a prevalent metabolic bone disorder marked by reduced bone mineral density and weakened bone structure, which increases the risk of fractures (1, 2). While osteoporosis is more common in women, the prevalence and associated risks in men have gained increasing attention in recent years (3). Epidemiological data show that the prevalence of osteoporosis in men is rising worldwide (4–6). With the aging population and changes in lifestyle, male osteoporosis has become a major public health concern. Studies report that 20% of white men are at risk for osteoporotic fractures, and their annual mortality rate from hip fractures is twice that of women. Although osteoporosis is less common in Black men than in white men, those diagnosed with the disease face similar fracture risks (7, 8). In the United States, over 2 million osteoporosis-related fractures occur each year, with experts predicting that the aging population will lead to a doubling of such fracture (9, 10). Consequently, effective treatment of male osteoporosis has become a critical issue in clinical practice.

A range of medications is available for the treatment of male osteoporosis, including anti-resorptive and bone-forming agents. Four commonly used medications—Alendronate, Risedronate, Teriparatide, and Denosumab—are widely employed in the clinical treatment of male osteoporosis, each exhibiting distinct efficacy and safety profiles. Alendronate, a bisphosphonate, primarily reduces bone resorption by inhibiting osteoclast activity, which improves bone mineral density. It is a first-line treatment for osteoporosis and is widely used in both men and women (11–13). Risedronate, another bisphosphonate, also inhibits bone resorption. Compared to other bisphosphonates, risedronate has higher bioavailability and a longer half-life, enabling a more sustained reduction in bone resorption (14–16). Teriparatide, a bone-forming agent, is a recombinant fragment of human parathyroid hormone. It enhances osteoblast function, stimulates bone formation, and increases bone mineral density. Unlike anti-resorptive drugs, Teriparatide reverses osteoporosis by stimulating new bone formation (17–19). Denosumab, a humanized monoclonal antibody, significantly reduces bone resorption by inhibiting osteoclastogenesis, improving bone density. Unlike bisphosphonates, it works through a different mechanism and is typically used for patients who cannot tolerate bisphosphonates (20–22).

These four medications are the most commonly used and well-researched treatments for male osteoporosis. Alendronate and risedronate, bisphosphonate-based anti-resorptive agents, are widely used in clinical practice. They inhibit osteoclast activity, reduce bone resorption, improve bone mineral density, and lower fracture risk, especially in the prevention and treatment of osteoporosis-related fractures (23–25). In contrast, teriparatide and denosumab represent novel therapeutic strategies targeting bone formation and resorption, respectively (26–28). Although several meta-analyses have examined this topic, three critical limitations persist in the current evidence: (1) existing studies have predominantly conducted pairwise comparisons, leaving the relative efficacy among all four first-line medications (ALE, RIS, TER, and DEN) systematically unaddressed; (2) previous analyses have often failed to incorporate both direct and indirect evidence when available; and (3) most focused exclusively on postmenopausal osteoporosis, with male populations being underrepresented. These gaps are particularly concerning given that clinical decision-making requires understanding the complete therapeutic landscape.

Network meta-analysis (NMA) provides an optimal solution to these limitations by: (1) enabling simultaneous comparison of all four drugs within a unified framework, (2) maximizing statistical power through integration of both direct and indirect evidence, and (3) allowing rank probability assessment of treatments through surface under the cumulative ranking (SUCRA) analysis. Leveraging these advantages, our study performs the first NMA specifically designed to compare ALE, RIS, TER, and DEN in male osteoporosis, with particular attention to fracture prevention efficacy and long-term safety profiles outcomes.

## 2 Materials and methods

### 2.1 Search strategy

This systematic review and network meta-analysis (NMA) was conducted in strict accordance with the PRISMA 2020 guidelines(Preferred Reporting Items for Systematic Reviews and Meta-Analyses) guidelines (29). To ensure transparency and standardization in the research process, the methodology was pre-registered on the international platform PROSPERO (registration number CRD42024599021). In our database selection for literature screening, in addition to the conventionally used PubMed and Web of Science, we further included the Cochrane Library, Scopus, and Embase to ensure comprehensive coverage of relevant studies, covering the period from the inception of each database up to June 2024. Only English-language studies were included. All search strategies and details are provided in the **Supplementary Materials**. Additionally, manual reference checking was performed by reviewing the references of published pairwise meta-analyses to ensure the inclusion of all high-quality studies, minimizing selection bias, and ensuring the comprehensiveness and reliability of the study results.

### 2.2 Inclusion and exclusion criteria

Eligible studies were selected based on the PICOS criteria (Population, Intervention, Comparison, Outcome, and Study Design). We included randomized controlled trials (RCTs) that evaluated the treatment of male osteoporosis with four medications. The specific PICOS criteria for study selection were as follows:

**Population**: Male patients with primary osteoporosis. Studies focusing on secondary osteoporosis (e.g., due to hypogonadism, corticosteroid-induced osteoporosis, cancer-related osteoporosis, or other systemic diseases) were excluded.

**Intervention**: Treatment with one of the four medications for primary osteoporosis based on random assignment.

**Comparison**: Comparison of the efficacy and safety of the four medications across different trial groups.

**Outcome**: Treatment outcomes were assessed by changes in lumbar spine, femoral neck and Total hip bone mineral density (BMD), as well as the percentage change in BMD, reflecting the efficacy of the medications.

1. Lumbar spine BMD: Refers to the density of minerals (mainly calcium) in the lumbar spine region, typically measured using dual-energy X-ray absorptiometry (DXA) (30). Lumbar BMD reflects the bone strength and health of the lower spine (lumbar region) and has predictive value for fracture risk (31).
2. Femoral neck BMD: Refers to the bone mineral density of the femoral neck, which is a common site for hip fractures. Measuring femoral neck BMD is crucial for assessing fracture risk and diagnosing osteoporosis (32).
3. Total hip BMD: Refers to the bone density in the entire hip region (including the femoral head, femoral shaft, and surrounding structures). This measurement provides a comprehensive reflection of hip bone strength and health, and hip fractures are among the most common fractures in the elderly (33).

Safety was evaluated by the number of patients reporting all adverse events and serious adverse events.

1. All adverse events: Any negative health reactions occurring during the clinical study or treatment process, including mild side effects such as headaches, nausea, or localized pain (34).
2. Serious adverse events: Health issues that pose a potential threat to life, such as fatal events, disability, hospitalization or prolonged hospitalization, or events resulting in significant health damage (35). All outcomes had a minimum follow-up period of 6 months.

**Study Design**: Only RCTs were included for meta-analysis. Non-original studies (e.g., case reports, reviews, letters to the editor, conference abstracts, opinion articles) and study protocols were excluded.

### 2.3 Data extraction and risk of bias assessment

Study selection and data collection were performed independently by two researchers (SJC and TY). First, the two reviewers screened titles and abstracts based on the predefined inclusion and exclusion criteria. Next, potentially eligible studies underwent full-text review. Any discrepancies in study selection were resolved through discussion, and if consensus could not be reached, a third researcher (RB) was consulted for arbitration. During data extraction, the following details were collected: the first author of the study, publication year, study design, country of origin, interventions, patient sample size, and follow-up duration. In case of any disagreements during the data extraction process, the third researcher (RB) was consulted to discuss and provide arbitration, ensuring the final consistency and accuracy of the data.

In terms of patient outcome assessment, we primarily focused on the percentage changes in lumbar spine, femoral neck, and bone mineral density (BMD) to evaluate the efficacy of the interventions. Additionally, the safety of the interventions was assessed by recording the number of patients experiencing all adverse events and serious adverse events following treatment. To address the issue of incomplete data in some studies, we followed the guidance provided in Section 6.5.2 of the Cochrane Intervention Review Handbook and used appropriate variance transformation or estimation methods (36). If the study reported mean differences and P-values, we calculated the standard error of the mean difference between groups using the formula provided in Section 6.5.2.3 and applied it in subsequent meta-analyses. In the absence of variance data, we assumed a conservative standard deviation of 30 for estimation. All these steps were carried out with strict adherence to scientific methodolog (36). Any discrepancies encountered during data extraction were resolved through discussion and consensus, with arbitration by a third researcher if necessary.

All included RCTs (randomized controlled trials) were assessed for risk of bias using the RoB 2 (a revised Cochrane risk-of-bias tool for randomized trials) to comprehensively evaluate potential biases in the study design and implementation process (37, 38).

### 2.4 Statistical analysis

We performed pairwise meta-analysis using Stata 18.0 software to evaluate the effect sizes and consistency across different studies. For network meta-analysis (NMA), we utilized R 4.3.1, combining the gemtc and BUGSnet packages to handle complex multi-treatment comparisons and assess the relative efficacy and safety of different treatment options (39, 40). All statistical analyses were conducted using Review Manager software (version 5.4), which is widely used in medical research for effective meta-analysis, forest plot generation, and bias risk assessment (More detailed information about the specific functions or models used is provided in **Supplementary Table S1**).

In terms of data synthesis, we selected appropriate statistical methods based on the type of data. For categorical variables, we used odds ratios (OR) to assess the relative risk between groups, describing the differences in the ratio of events occurring between the treatment and control groups. For continuous variables, we used mean differences (MD) to analyze the effect sizes between groups and reveal the effects of the treatment interventions (41).

## 3 Results

### 3.1 Literature search and study selection

**Figure 1:**
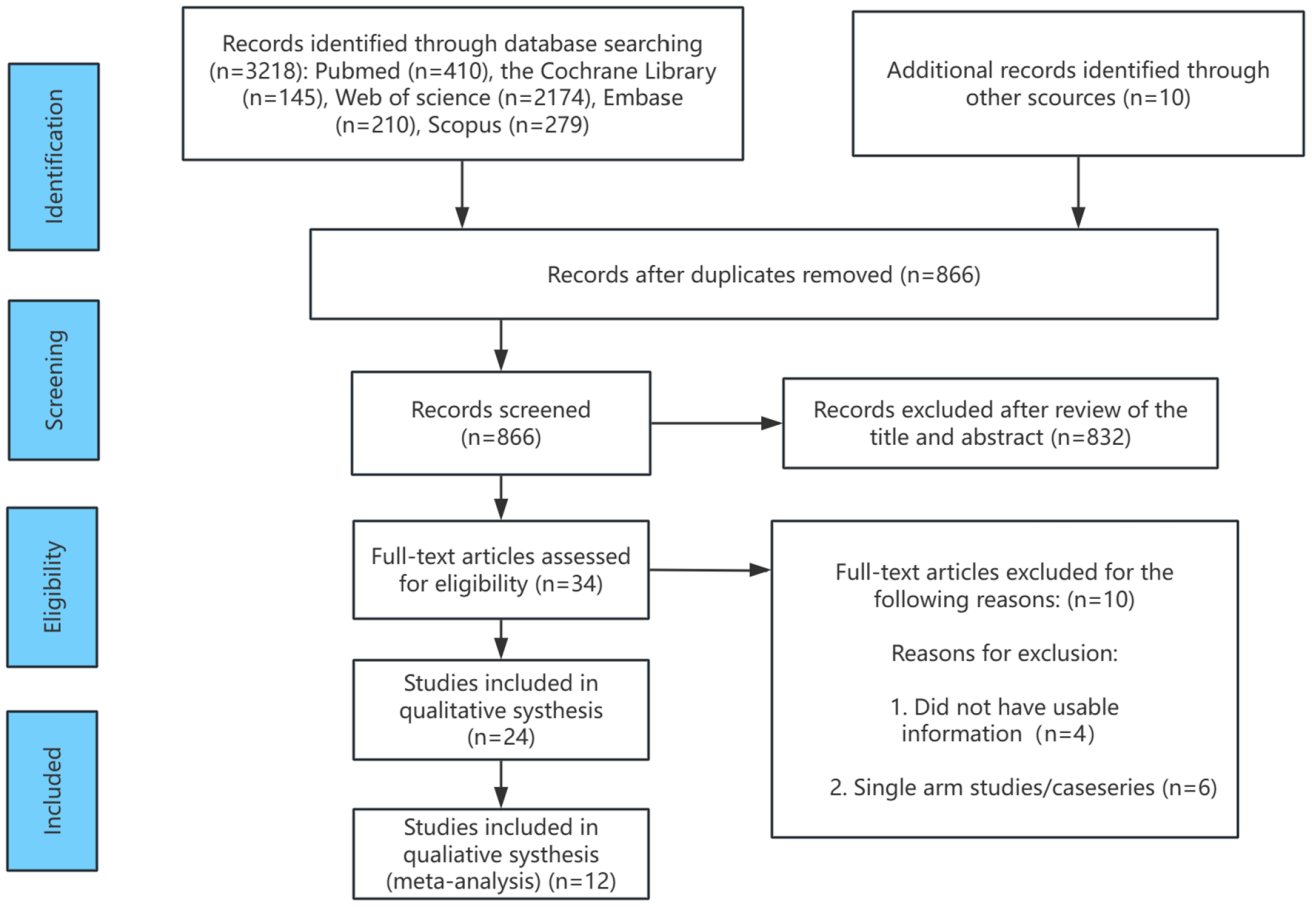
Flow chart of the selection process for relative studies in meta-analysis.

The flowchart outlining the RCT selection process is presented in **Figure 1**. A total of 2,729 records were identified during the literature search for review. Of these, 1,863 records were excluded due to duplication (i.e., identical citations across multiple databases), leaving 866 unique records for further evaluation. Following full-text review and application of the inclusion criteria, additional studies were excluded. For instance, Ringe 2001 (42) and Ringe 2004 (43) were two RCTs conducted by the same research group at different time points. To prevent data duplication, the 2001 publication was excluded. Wallach 2000 (44), which focused on male osteoporosis following high-dose corticosteroid therapy, was excluded as it did not align with the focus of this study. Similarly, Shimon 2005 (45) and Smith 2009 (46) were excluded as they focused on osteoporosis in men with androgen-deficient hypogonadism and those undergoing androgen deprivation therapy for prostate cancer, respectively, which did not align with the clinical population in this study. Furthermore, Saag 2019 (47) and Nakamura 2014 (48) were excluded due to insufficient data on male patients or unclear gender differentiation. Following these exclusions, 12 RCTs involving 1,935 participants were included in the analysis, with four treatment options (alendronate, risedronate, teriparatide, and denosumab), along with placebo and alfacalcidol. These studies formed the basis for the subsequent network meta-analysis.

**Figure 2:**
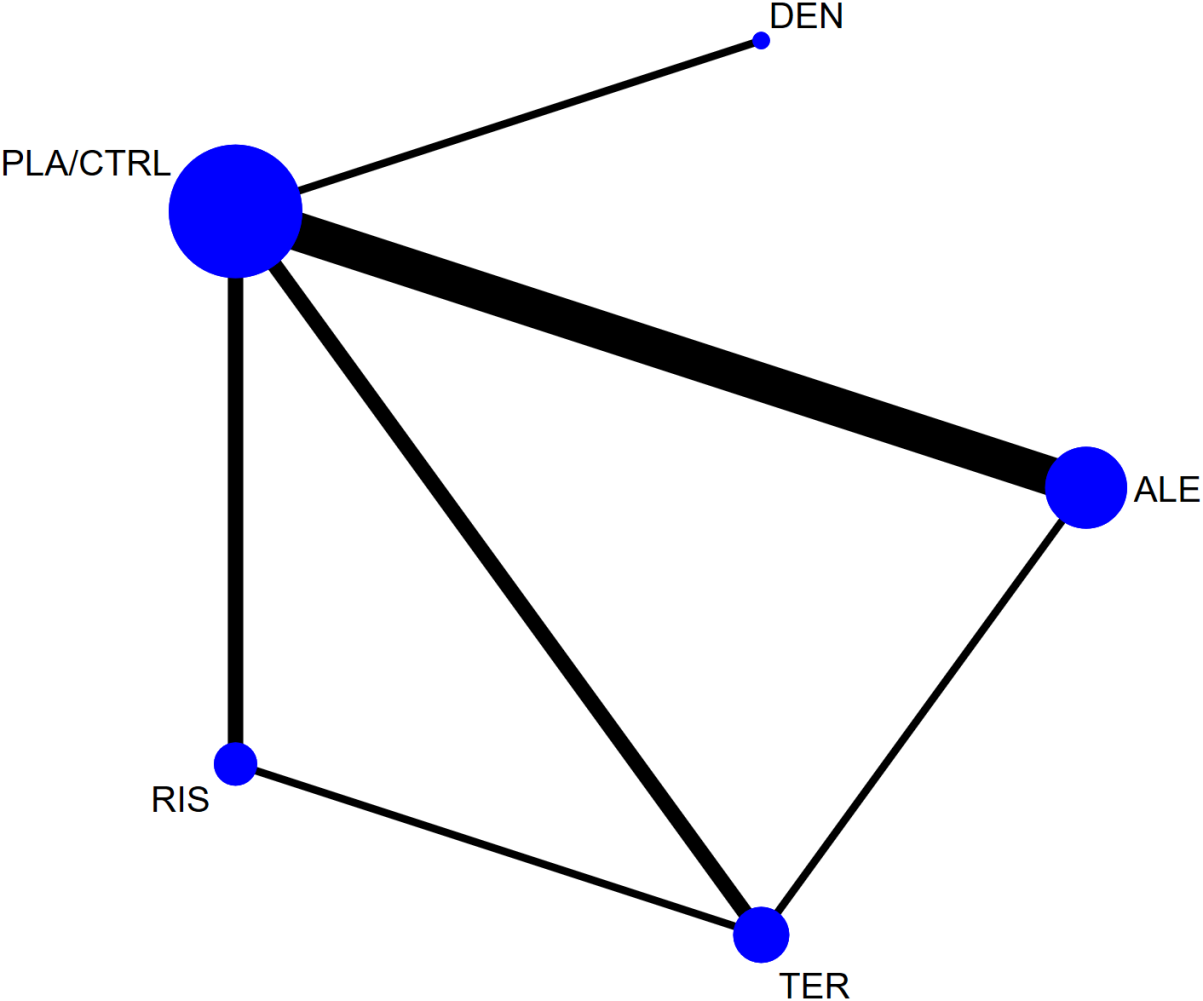
The network plot of all trials (ALE, alendronate; RIS, risedronate; TER, Teriparatide; DEN, denosumab; PLA/CTRL, placebo/control).

**Figure 2** shows the network diagram for all studies. The node-splitting method revealed no significant inconsistency (P > 0.05). Statistical analysis indicated that both the consistency and inconsistency models demonstrated good coherence for all outcomes in **Supplementary Table S2**. The follow-up period for primary outcomes (lumbar spine, femoral neck, and total hip BMD) was 12 months, with fewer studies reporting longer or shorter durations. To ensure the reliability of the results, we used closest to 12 months follow-up data for analysis. **Supplementary Figure S1** shows the forest plot results for all outcomes. **Supplementary Figure S2** shows the network diagram for all outcomes. The size of the nodes corresponds to the number of participants in each treatment group, and the thickness of the lines between nodes reflects the number of studies comparing the treatments. **Supplementary Figure S3** presents the funnel plots for bias analysis of all outcomes. Visual inspection of the funnel plots indicated no publication bias in the included studies.

### 3.2 Study characteristics

**Table 1** summarizes the 12 randomized controlled trials (RCTs) included in the network meta-analysis (NMA), along with their key characteristics (43, 49–59). The studies were published between 2000 and 2021, with male participant numbers ranging from 19 to 290, and study durations spanning 1 to 3 years. Most studies compared treatment regimens to placebo and/or calcium and vitamin D, while some also included alfacalcidol as a comparator. Direct comparisons among the four treatment options were limited. Excluding multicenter studies, the majority of relevant studies were from Germany, the United States, and China, with two studies from each included in the analysis. The dosages of the included medications reflect those commonly used in clinical practice: risedronate (35 mg weekly or 5 mg daily orally), alendronate (70 mg weekly or 10 mg daily orally), teriparatide (20 μg subcutaneously daily), and denosumab (60 mg subcutaneously every 6 months). These regimens were widely used in the included studies, forming the basis for treatment comparisons.

**Table 1:** The main features of the articles.

Author, year	Study design	Country	Treatment	Comparator	Background treatment	Group/Patient	Length of interv
Boonen 2009 (55)	RCT	Multicenter study	Risedronate 35 mg, daily oral administration	Placebo	Ca (1 g) and vit D (400–500 IU), twice daily	G1: 191 G2: 93	104 weeks (2 years)
Gonnelli 2003 (50)	RCT	Italy	Alendronate 10 mg, oral daily administration	No placebo	Ca (1000 mg) daily oral administration	G1: 39 G2: 38	156 weeks (3-years)
Hwang 2010 (54)	RCT	China	Alendronate 70 mg, oral weekly administration	No placebo	Ca and Vit D supplement, daily oral administration	G1: 23 G2: 23	24 weeks
Miller 2004 (59)	RCT	USA, Multicentre	Alendronate 70 mg, weekly oral administration	Placebo	Ca as carbonate (500 mg) and Vit D (200 IU), daily oral administration	G1: 109 G2: 58	52 weeks (1 year)
Orwoll 2000 (49)	RCT	20 centers in the United States and 10 other countries	Alendronate 10 mg, daily oral administration	Placebo	Ca (500 mg) and Vit D (400 IU), daily oral administration	G1:146 G2: 95	104 weeks (2 years)
Orwoll 2003 (53)	RCT	37 centers in 11 countries	Teriparatide 20ug, subcutaneous daily injection	1: Teriparatide 40ug, subcutaneous daily injection 2: Placebo	Ca (1000 mg) and Vit D (400–1200 IU), daily oral administration	G1: 151 G2: 139 G3: 147	52 weeks (1 year)
Orwoll 2012 (56)	RCT	Multicentre study (North America and Europe)	Denosumab 60 mg, sub cutaneous injection every 6 months (q6m)	Placebo	Ca (≥ 1 g) and Vit D (≥ 800 IU), daily oral administration	G1: 121 G2: 121	52 weeks (1 year)
Qi 2021 (52)	RCT	China	Teriparatide 20 µg/day, daily subcutaneous injection	Alendronate 10 mg/day, oral daily administration	Ca and Vit D (dose not provided), daily oral administration	G1: 50 G2: 50	52 weeks (1 year)
Ringe 2009 (57)	RCT	Germany	Risedronate 5 mg, oral daily administration	Daily alfacalcidol (1 microg)	Ca (1,000 mg) daily and Vit D (800 IU), daily oral administration	G1: 158 G2: 158	104 weeks (2 years)
Walker 2013 (51)	RCT	Columbia	Risedronate oral 35 mg, weekly oral administration	1: Teriparatide daily subcutaneous injection 20 µg 2: Combination of both	Ca (500 mg) and vit D (400 IU), daily oral administration	G1: 10 G2: 9 G3: 10	78 weeks (18 months)
Kaufman 2004 (58)	RCT	37 study sites in 11 countries	Teriparatide 20ug, subcutaneous daily injection	1: Teriparatide 40ug, subcutaneous daily injection 2: Placebo	Supplemental calcium (1,000 mg daily) and vitamin D (400–1,200 IU daily)	G1: 22 G2: 20 G3: 37	18 months
Ringe 2004 (43)	RCT	Germany	Alendronate 10 mg, oral daily administration	Alfacalcidol (1 μg daily)	Supplemental calcium (500 mg daily)	G1: 68 G2: 66	3 years

### 3.3 Study quality and potential sources of bias

**Figure 3:**
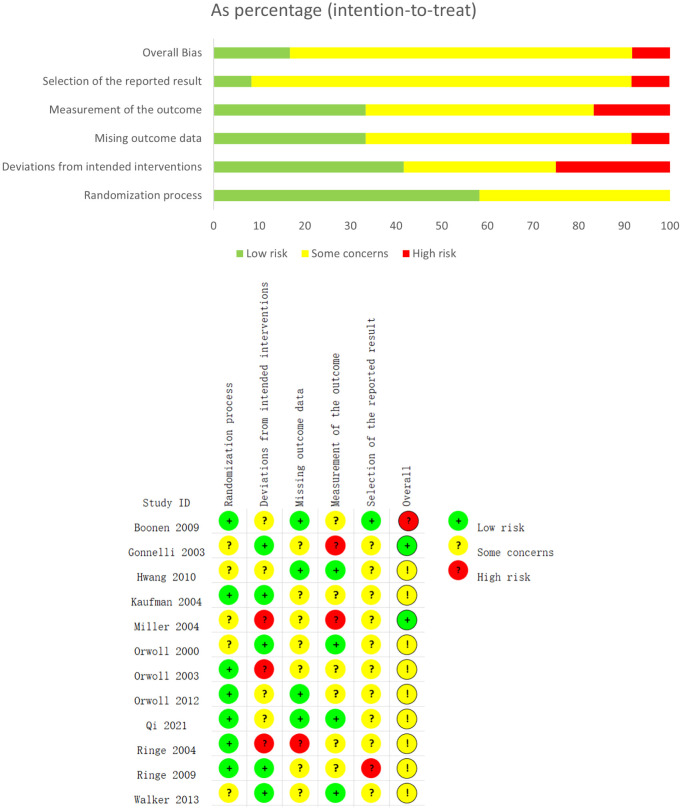
The network plot of all trials. Risk of bias summary for RCTs: Reviewers' judgments about each risk of bias item per included study.

**Figure 3** shows the results of the risk of bias assessment for the included studies, conducted according to the Cochrane Collaboration’s guidelines. The assessment evaluates potential biases in the included studies, focusing specifically on random sequence generation, allocation concealment, and the completeness of outcome reporting. Overall, most studies inadequately reported the methods for random sequence generation and allocation concealment, particularly the details of the randomization process. Many studies also failed to clearly report whether all pre-specified outcomes were presented as per the study protocol, a key factor in assessing outcome reporting bias. Due to methodological opacity, we could not definitively determine whether these studies had a “low” or “high” risk of bias in random sequence generation, allocation concealment, and outcome reporting. As a result, most studies were categorized as having an “unclear risk” of bias for selection bias (due to improper randomization) and reporting bias (due to incomplete reporting of pre-specified outcomes).

#### 3.3.1 Percentage change in lumbar spine bone mineral density

**Figure 4:**
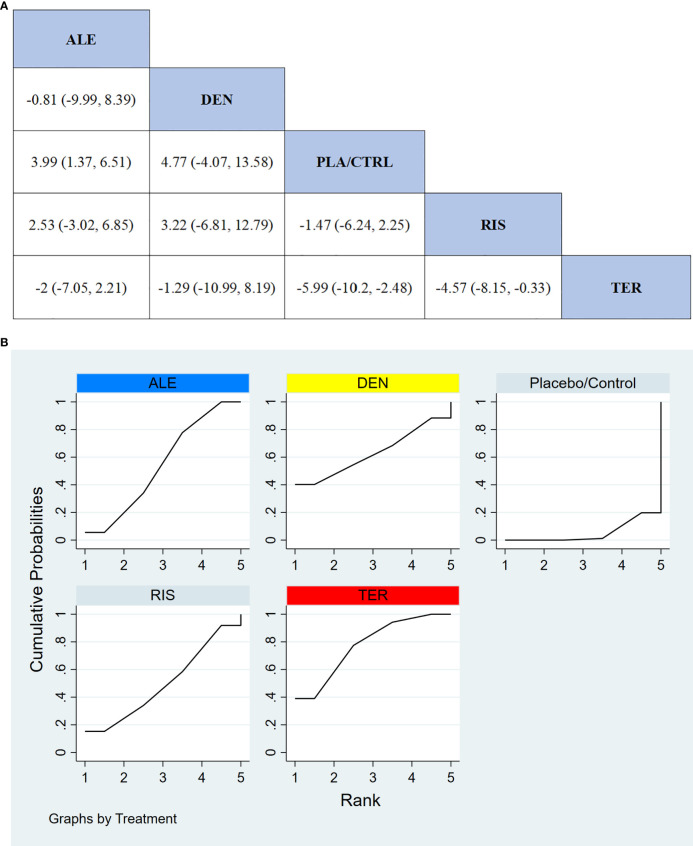
**(A)** The results of League table for Lumbar spine BMD. **(B)** Ranking the probability of Lumbar spine BMD percentage change.

In 9 RCTs involving a total of 1,587 participants, we compared the effects of different treatments on lumbar spine BMD. The results showed that the TER treatment group significantly outperformed other drugs in terms of increasing lumbar spine BMD. **Figure 4A** displays the specific differences between treatment groups. Further analysis revealed that, apart from TER, the differences in BMD outcomes between the other treatment groups did not reach statistical significance.

#### 3.3.2 Ranking probability of lumbar spine BMD improvement

To further quantify the effectiveness of different treatments in improving lumbar spine BMD, we performed a cumulative ranking curve analysis (SUCRA). **Figure 4B** shows the results for lumbar spine BMD improvement, where red indicates the highest rank, yellow represents the second rank, and blue represents the third rank. The ranking results revealed significant differences in the effectiveness of the drugs in improving lumbar spine BMD, with higher rankings indicating better treatment effects. According to the SUCRA analysis, the probabilities for improving lumbar spine BMD from highest to lowest were as follows: TER (77.7%), DEN (62.9%), ALE (54.3%), RIS (49.9%), and PLA/CTRL (5.2%).

#### 3.3.3 Percentage change in femoral neck BMD

**Figure 5:**
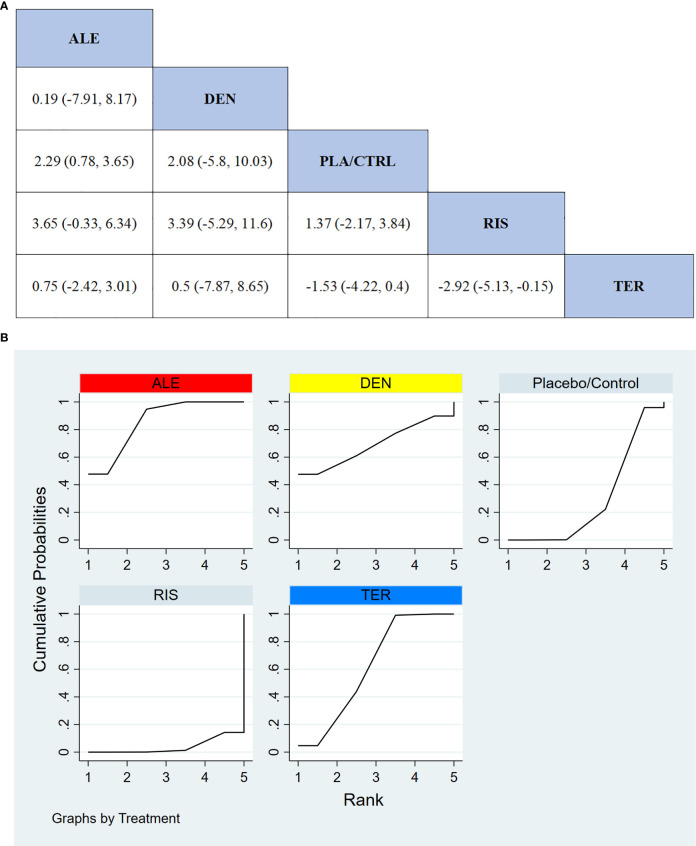
**(A)** The results of League table for Femoral neck BMD. **(B)** Ranking the probability of Femoral neck BMD percentage change.

In 9 RCTs with a total of 1,594 participants, we compared the effects of different treatments on femoral neck BMD. The results indicated that the ALE treatment group significantly outperformed other treatment options in improving femoral neck BMD. **Figure 5A** shows the specific differences between the treatment groups.

#### 3.3.4 Ranking probability of femoral neck BMD improvement

To further quantify the effectiveness of different treatments in improving femoral neck BMD, we used the cumulative ranking curve (SUCRA) method to rank the treatments. **Figure 5B** presents the results for femoral neck BMD improvement. Based on the SUCRA analysis, the probabilities for improving femoral neck BMD, from highest to lowest, were as follows: ALE (85.6%), DEN (69.0%), TER (61.9%), PLA/CTRL (29.6%), and RIS (3.9%).

#### 3.3.5 Percentage change in total hip BMD

**Figure 6:**
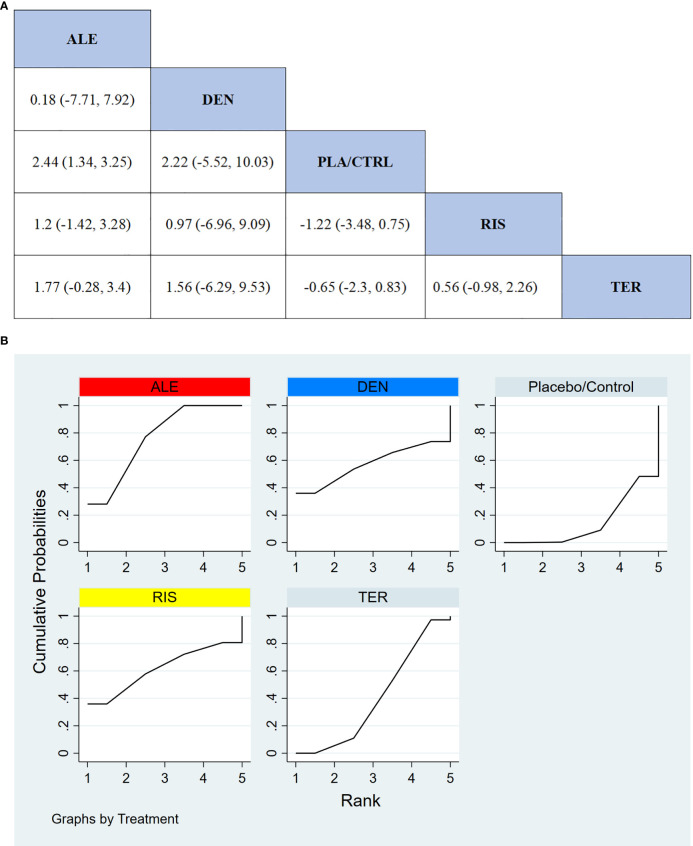
**(A)** The results of League table for Total hip BMD. **(B)** Ranking the probability of Total hip BMD percentage change.

In 8 RCTs involving a total of 1,310 participants, we compared the effects of different treatments on total hip BMD. The results demonstrated that the ALE treatment group significantly outperformed other treatment groups in improving total hip BMD. **Figure 6A** displays the specific differences between treatment groups. Further analysis showed that, aside from ALE, the differences in effects between the other treatment groups did not reach statistical significance.

#### 3.3.6 Ranking probability of total Hip BMD improvement

To further quantify the effectiveness of different treatments in improving total hip BMD, we employed the cumulative ranking curve (SUCRA) method. **Figure 6B** presents the results for total hip BMD improvement. According to the SUCRA analysis, the probabilities for improving total hip BMD, from highest to lowest, were as follows: ALE (88.0%), DEN (60.5%), RIS (57.5%), TER (35.5%), and PLA/CTRL (8.6%).

#### 3.3.7 All adverse events

**Figure 7:**
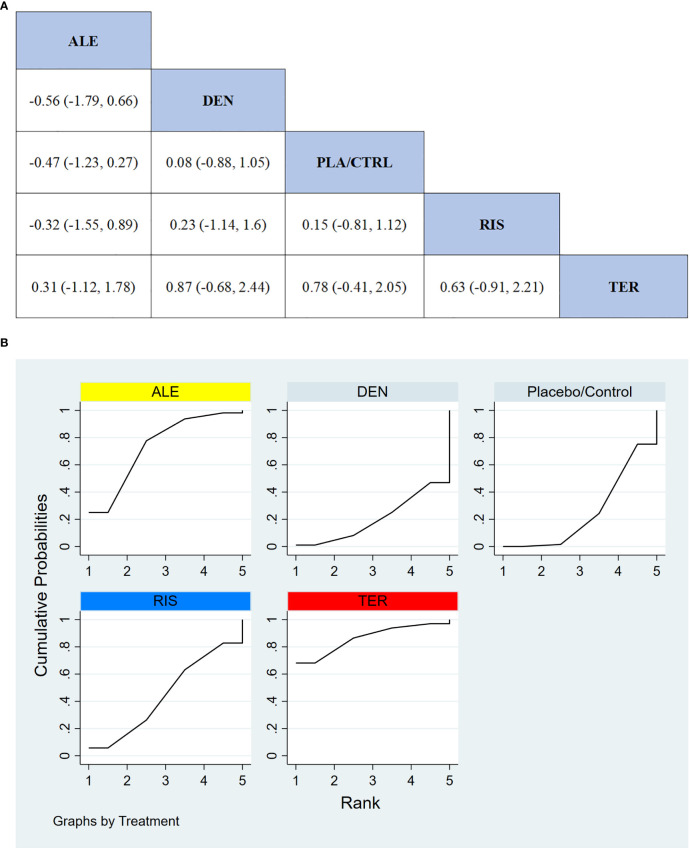
**(A)** The results of League table for all adverse events. **(B)** Ranking the probability of all adverse events.

In 5 RCTs involving a total of 590 participants, we compared the all adverse events of different treatments. The results indicated that the TER treatment group had significantly better safety compared to other treatment groups. **Figure 7A** shows the specific differences between treatment groups.

#### 3.3.8 Ranking probability of all adverse events

To further quantify the safety of different treatments in terms of all adverse events, we used the cumulative ranking curve (SUCRA) method. **Figure 7B** presents the results for all adverse events. Based on the SUCRA analysis, the probabilities for safety in terms of all adverse events, from highest to lowest, were as follows: TER (86.4%), ALE (73.6%), RIS (44.5%), PLA/CTRL (25.3%), and DEN (20.3%).

#### 3.3.9 Serious adverse events

**Figure 8:**
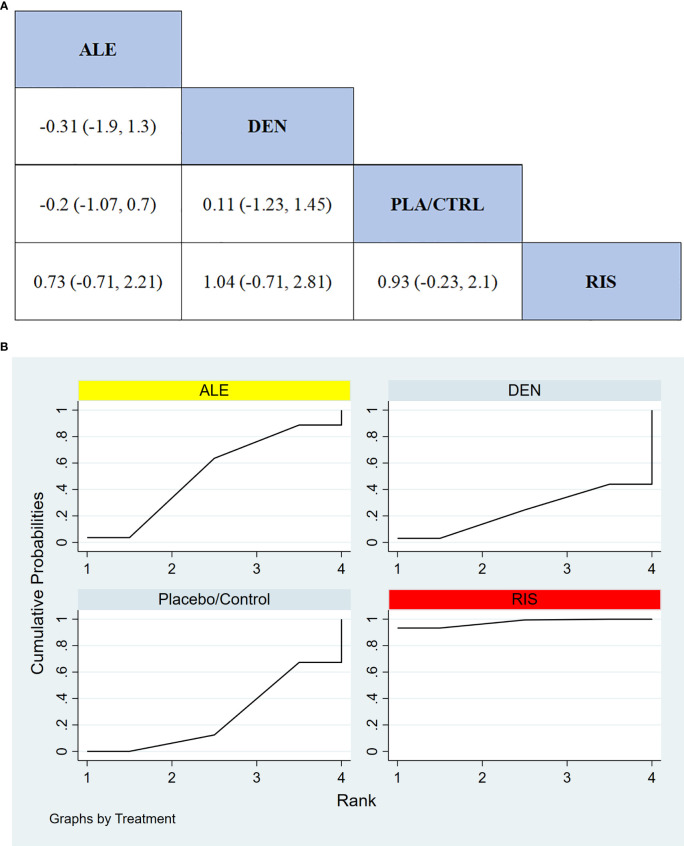
**(A)** The results of League table for serious adverse events. **(B)** Ranking the probability of serious adverse events.

In 4 RCTs involving a total of 150 participants, we compared the serious adverse events between different treatments (TER was not included due to insufficient data). The results showed that the RIS treatment group had significantly better safety compared to other treatment groups in terms of serious adverse events. **Figure 8A** displays the specific differences between the treatment groups.

#### 3.3.10 Ranking probability of serious adverse events

To further quantify the safety of different treatments in terms of serious adverse events, we used the cumulative ranking curve (SUCRA) method. **Figure 8B** presents the results for serious adverse events. According to the SUCRA analysis, the ranking probabilities for safety in terms of serious adverse events, from highest to lowest, were as follows: RIS (97.6%), ALE (52.0%), PLA/CTRL (26.6%), and DEN (23.9%).

## 4 Discussion

This systematic review and network meta-analysis (NMA) provides a comprehensive evaluation of the efficacy and safety of various drugs (TER, ALE, DEN, and RIS) in the treatment of male osteoporosis. The results showed significant differences in the effects of these four drugs on bone mineral density (BMD) improvement across different skeletal sites. TER showed the greatest efficacy in improving lumbar spine BMD, significantly outperforming the other treatments. In contrast, ALE exhibited the strongest effects in enhancing femoral neck and total hip BMD, demonstrating a clear advantage at these sites. These findings suggest that TER and ALE may operate through different mechanisms at specific bone sites, with TER being particularly effective for the lumbar spine, while ALE has stronger effects on the femoral neck and hip.

Regarding safety, TER demonstrated the best safety profile, with the lowest incidence of all adverse events and no significant serious adverse events. This makes TER a suitable option for patients requiring long-term treatment and prioritizing safety. RIS also exhibited a favorable safety profile, particularly in preventing serious adverse events, with the lowest occurrence of severe adverse events. However, RIS was less effective than TER and ALE in improving BMD, suggesting that it may be better suited for patients who prioritize safety over BMD improvement.

The cumulative ranking curve (SUCRA) analysis indicated that TER ranked highest for improving lumbar spine BMD and exhibited the best safety profile, making it a comprehensive treatment option. ALE demonstrated the greatest improvements in femoral neck and total hip BMD, but its safety was slightly inferior to TER’s. Thus, the effectiveness and safety of ALE should be balanced when choosing a treatment. Although DEN improved lumbar spine and femoral neck BMD, it exhibited poorer safety, particularly regarding all adverse events. Caution is advised when using this drug. RIS excelled in preventing serious adverse events but showed weaker BMD improvement, especially in the lumbar spine and femoral neck. It is therefore more suitable for patients with high safety requirements but less concern for maximizing BMD enhancement. These findings corroborate the results from Charlotte Beaudart et al. (60) and Smita Nayak et al. (61).

In recent years, the rising prevalence of male osteoporosis has led to increased clinical research, particularly systematic reviews and network meta-analyses (NMAs). Previous NMAs have primarily focused on other aspects of interventions. For example, Chen et al. (62) conducted an NMA to assess the effects of eight drugs on male bone density, offering a hierarchical analysis. The main findings of their study focused on lumbar spine BMD and fracture incidence but did not analyze femoral neck and total hip BMD or report all adverse events, including serious ones. In contrast, traditional pairwise meta-analyses have compared the effects of different treatments. For example, Charlotte Beaudart et al. (60) and Smita Nayak et al. (61) conducted pairwise meta-analyses. Beaudart et al. concluded that drugs for female osteoporosis are also beneficial for males, while Nayak et al. found that bisphosphonates significantly reduce the risk of vertebral, and possibly non-vertebral, fractures in male osteoporosis patients. Although these studies provided valuable insights, they were limited to pairwise comparisons of two treatment options and did not comprehensively assess the relative efficacy and safety of the four drugs. This study thus employed network meta-analysis (NMA) to comprehensively rank the efficacy and safety of four drugs (TER, ALE, DEN, and RIS) in treating male osteoporosis. To the best of our knowledge, this is the first large-scale NMA to compare the therapeutic effects of TER, ALE, DEN, and RIS for male osteoporosis.

This network meta-analysis (NMA) has several advantages: (1) It includes 12 studies with a total of 1,935 participants, all randomized controlled trials (RCTs), ensuring data reliability; (2) The statistical results demonstrate good consistency, reflecting the stability of the methods and the reproducibility of the conclusions; (3) By indirectly comparing the effects of different treatments, this study offers a comprehensive evaluation of pharmacological management for male osteoporosis. However, this study has several limitations: (1) Some treatment drugs lacked direct head-to-head comparisons, limiting pairwise analysis between certain drugs; (2) Some safety data (e.g., fracture incidence) were incomplete and not included in the analysis; (3) The studies spanned a long period (2000–2021), which may have led to variations in study design, patient characteristics, and data collection methods, potentially affecting the quality of the results.(4) Subgroup analysis can be more helpful in understanding whether certain patient populations benefit more or less from specific treatments. However, the data from included literature are incomplete in aspects such as the severity of osteoporosis, comorbidities, and various demographic factors. This necessitates further improvement in future research. Future research should focus on conducting high-quality RCTs, especially direct comparison studies of these drugs, to further validate their efficacy and safety in treating male osteoporosis. Such studies will yield more reliable results and enhance our understanding of the clinical value of different treatment strategies for male osteoporosis.

## 5 Conclusion

Based on our network meta-analysis, TER emerges as the most balanced therapeutic option for male osteoporosis, demonstrating superior efficacy in improving lumbar spine BMD alongside the most favorable safety profile. ALE is the optimal choice for enhancing BMD at the femoral neck and total hip, though its slightly higher risk of adverse events warrants careful consideration in patients prioritizing safety. RIS, while less effective for BMD improvement, may be suitable for patients with heightened safety concerns due to its low incidence of serious adverse events. DEN showed significant BMD benefits but carried the highest risk of adverse events, necessitating individualized risk-benefit assessment. These findings underscore the need for personalized treatment strategies tailored to skeletal site-specific deficits and patient tolerance for adverse events. Future studies should explore combination therapies or novel agents to further optimize the efficacy-safety balance in male osteoporosis.

## Data Availability

The original contributions presented in the study are included in the article/**Supplementary Material**. Further inquiries can be directed to the corresponding author.
