# Supplementary material for: The efficacy and safety of denosumab, risedronate, alendronate and teriparatide to treat male osteoporosis: a systematic review and bayesian network meta-analysis
Source: Front Endocrinol (Lausanne). 2025 Jun 19;16:1579101. doi: 10.3389/fendo.2025.1579101 (PMC12221909; doi:10.3389/fendo.2025.1579101)

**Appendix.**

**Supplementary data**

**Search Strategy;**

**PUBMED （410）**

(((((((((((((((((((Osteoporosis[MeSH Terms]) OR (Osteoporoses[Title/Abstract])) OR (Osteoporosis, Age-Related[Title/Abstract])) OR (Osteoporosis, Age Related[Title/Abstract])) OR (Age-Related Osteoporosis[Title/Abstract])) OR (Age-Related Osteoporoses[Title/Abstract])) OR (Age Related Osteoporosis[Title/Abstract])) OR (Osteoporoses, Age-Related[Title/Abstract])) OR (Bone Loss, Age-Related[Title/Abstract])) OR (Age-Related Bone Loss[Title/Abstract])) OR (Age-Related Bone Losses[Title/Abstract])) OR (Bone Loss, Age Related[Title/Abstract])) OR (Bone Losses, Age-Related[Title/Abstract])) OR (Osteoporosis, Senile[Title/Abstract])) OR (Osteoporoses, Senile[Title/Abstract])) OR (Senile Osteoporoses[Title/Abstract])) OR (Senile Osteoporosis[Title/Abstract])) OR (Osteoporosis, Involutional[Title/Abstract])) AND (men[Title/Abstract])) AND (((((((((((((((((((((((((((((((Alendronate[MeSH Terms]) OR (Aminohydroxybutane Bisphosphonate[Title/Abstract])) OR (4-Amino-1-Hydroxybutylidene 1,1-Biphosphonate[Title/Abstract])) OR (MK-217[Title/Abstract])) OR (MK217[Title/Abstract])) OR (MK 217[Title/Abstract])) OR (Fosamax[Title/Abstract])) OR (Alendronate Sodium[Title/Abstract])) OR (Alendronate Monosodium Salt, Trihydrate[Title/Abstract])) OR (Risedronic Acid[MeSH Terms])) OR (Bisphosphonate Risedronate Sodium[Title/Abstract])) OR (Risedronate Sodium, Bisphosphonate[Title/Abstract])) OR (Sodium, Bisphosphonate Risedronate[Title/Abstract])) OR (Risedronate Sodium[Title/Abstract])) OR (Actonel[Title/Abstract])) OR (Risedronic Acid, Monosodium Salt[Title/Abstract])) OR (Risedronate[Title/Abstract])) OR (1-Hydroxy-2-(3-pyridyl)ethylidene diphosphonate[Title/Abstract])) OR (Atelvia[Title/Abstract])) OR (2-(3-pyridinyl)-1-hydroxyethylidene-bisphosphonate[Title/Abstract])) OR (2-(3-pyridinyl)-1-hydroxyethylidenebisphosphonate[Title/Abstract])) OR (Teriparatide[MeSH Terms])) OR (hPTH (1-34[Title/Abstract]))) OR (Human Parathyroid Hormone (1-34[Title/Abstract]))) OR (Parathar[Title/Abstract])) OR (Teriparatide Acetate[Title/Abstract])) OR (Forteo[Title/Abstract])) OR (Denosumab[MeSH Terms])) OR (AMG 162[Title/Abstract])) OR (Xgeva[Title/Abstract])) OR (Prolia[Title/Abstract]))

**Cochrane Library （145）**

#1 MeSH descriptor: [Osteoporosis] explode all trees

#2 men

#3 #1 and #2

#4 MeSH descriptor: [Alendronate] explode all trees

#5 MeSH descriptor: [Risedronic Acid] explode all trees

#6 MeSH descriptor: [Teriparatide] explode all trees

#7 MeSH descriptor: [Denosumab] explode all trees

#8 #4 or #5 or #6 or #7

#9 #3 and #8

**Web of science （2174）**

1: (((((((((((((((((TS=(Osteoporosis)) OR TS=(Osteoporoses)) OR TS=(Osteoporosis, Age-Related)) OR TS=(Osteoporosis, Age Related)) OR TS=(Age-Related Osteoporosis)) OR TS=(Age-Related Osteoporoses)) OR TS=(Age Related Osteoporosis)) OR TS=(Osteoporoses, Age-Related)) OR TS=(Bone Loss, Age-Related)) OR TS=(Age-Related Bone Loss)) OR TS=(Age-Related Bone Losses)) OR TS=(Bone Loss, Age Related)) OR TS=(Bone Losses, Age-Related)) OR TS=(Osteoporosis, Senile)) OR TS=(Osteoporoses, Senile)) OR TS=(Senile Osteoporoses)) OR TS=(Senile Osteoporosis)) OR TS=(Osteoporosis, Involutional)

2: TS=(men)

3: #1 AND #2

4: (((((((((((((((((((((((((((((((TS=(Alendronate)) OR TS=(Aminohydroxybutane Bisphosphonate)) OR TS=(4-Amino-1-Hydroxybutylidene 1,1-Biphosphonate)) OR TS=(MK-217)) OR TS=(MK217)) OR TS=(MK 217)) OR TS=(Fosamax)) OR TS=(Alendronate Sodium)) OR TS=(Alendronate Monosodium Salt, Trihydrate)) OR TS=(Risedronic Acid)) OR TS=(Bisphosphonate Risedronate Sodium)) OR TS=(Risedronate Sodium, Bisphosphonate)) OR TS=(Sodium, Bisphosphonate Risedronate)) OR TS=(Risedronate Sodium)) OR TS=(Actonel)) OR TS=(Risedronic Acid, Monosodium Salt)) OR TS=(Risedronate)) OR TS=(1-Hydroxy-2-(3-pyridyl)ethylidene diphosphonate)) OR TS=(Atelvia)) OR TS=(2-(3-pyridinyl)-1-hydroxyethylidene-bisphosphonate)) OR TS=(2-(3-pyridinyl)-1-hydroxyethylidenebisphosphonate)) OR TS=(Teriparatide)) OR TS=(hPTH (1-34))) OR TS=(Human Parathyroid Hormone (1-34))) OR TS=(Parathar)) OR TS=(Teriparatide Acetate))) OR TS=(Forteo)) OR TS=(Denosumab)) OR TS=(AMG 162)) OR TS=(Xgeva)) OR TS=(Prolia)

5: #3 AND #4

**SCOPUS (279)**

( RCT ) AND ( ( {Osteoporosis} AND {men} ) AND ( {Alendronate} OR {Risedronic Acid} OR {Teriparatide} OR {Denosumab} OR "Aminohydroxybutane Bisphosphonate" OR "Fosamax" OR "Alendronate Sodium" OR "Bisphosphonate Risedronate Sodium" OR "Actonel" OR "Atelvia" OR "Teriparatide Acetate" OR "Prolia" ) ) AND PUBYEAR > 1998 AND PUBYEAR < 2025 AND ( LIMIT-TO ( SUBJAREA , "MEDI" ) )

**EMBASE (210)**

#4. #1 AND #2 AND #3

#3. '4 amino 1 hydroxy 1, 1 butanebisphosphonic

acid'/exp OR '4 amino 1 hydroxy 1, 1

butanebisphosphonic acid' OR '4 amino 1 hydroxy

1, 1 butanediphosphonic acid'/exp OR '4 amino 1

hydroxy 1, 1 butanediphosphonic acid' OR '4 amino

1 hydroxybutane 1, 1 diphosphonate'/exp OR '4

amino 1 hydroxybutane 1, 1 diphosphonate' OR '4

amino 1 hydroxybutane 1, 1 diphosphonic acid'/exp

OR '4 amino 1 hydroxybutane 1, 1 diphosphonic

acid' OR '4 amino 1 hydroxybutylidene 1, 1

bisphosphonate'/exp OR '4 amino 1

hydroxybutylidene 1, 1 bisphosphonate' OR '4

amino 1 hydroxybutylidene 1, 1 bisphosphonic

acid'/exp OR '4 amino 1 hydroxybutylidene 1, 1

bisphosphonic acid' OR '4 amino 1

hydroxybutylidene 1, 1 diphosphonate'/exp OR '4

amino 1 hydroxybutylidene 1, 1 diphosphonate' OR

'4 amino 1 hydroxybutylidene 1, 1 diphosphonic

acid'/exp OR '4 amino 1 hydroxybutylidene 1, 1

diphosphonic acid' OR 'adronat'/exp OR 'adronat'

OR 'alenato'/exp OR 'alenato' OR 'alend'/exp OR

'alend' OR 'alendronate'/exp OR 'alendronate' OR

'alendronate sodium'/exp OR 'alendronate sodium'

OR 'alendronate sodium trihydrate'/exp OR

'alendronate sodium trihydrate' OR 'alendros'/exp

OR 'alendros' OR 'alovell'/exp OR 'alovell' OR

'arendal'/exp OR 'arendal' OR 'bifemelan'/exp OR

'bifemelan' OR 'bifosa'/exp OR 'bifosa' OR

'binosto'/exp OR 'binosto' OR 'bonalon'/exp OR

'bonalon' OR 'bonapex'/exp OR 'bonapex' OR

'bonasol'/exp OR 'bonasol' OR 'defixal'/exp OR

'defixal' OR 'dronal'/exp OR 'dronal' OR

'endronax'/exp OR 'endronax' OR 'eucalen'/exp OR

'eucalen' OR 'ex 101'/exp OR 'ex 101' OR

'ex101'/exp OR 'ex101' OR 'fixopan'/exp OR

'fixopan' OR 'fosalan'/exp OR 'fosalan' OR

'fosamac'/exp OR 'fosamac' OR 'fosamax'/exp OR

'fosamax' OR 'fosmin'/exp OR 'fosmin' OR

'fosval'/exp OR 'fosval' OR 'g 704650'/exp OR 'g

704650' OR 'g704650'/exp OR 'g704650' OR

'genalen'/exp OR 'genalen' OR 'gth 42'/exp OR

'gth 42' OR 'gth 42j'/exp OR 'gth 42j' OR 'gth

42v'/exp OR 'gth 42v' OR 'gth 42w'/exp OR 'gth

42w' OR 'gth42'/exp OR 'gth42' OR 'gth42j'/exp OR

'gth42j' OR 'gth42v'/exp OR 'gth42v' OR

'gth42w'/exp OR 'gth42w' OR 'l 670452'/exp OR 'l

670452' OR 'l670452'/exp OR 'l670452' OR 'labr

312'/exp OR 'labr 312' OR 'labr312'/exp OR

'labr312' OR 'marvil'/exp OR 'marvil' OR

'maxibone'/exp OR 'maxibone' OR 'maxibone 70'/exp

OR 'maxibone 70' OR 'mk 0217'/exp OR 'mk 0217' OR

'mk 217'/exp OR 'mk 217' OR 'mk0217'/exp OR

'mk0217' OR 'mk217'/exp OR 'mk217' OR

'neobon'/exp OR 'neobon' OR 'oncalst'/exp OR

'oncalst' OR 'onclast'/exp OR 'onclast' OR

'osdron'/exp OR 'osdron' OR 'osdronat'/exp OR

'osdronat' OR 'oseotenk'/exp OR 'oseotenk' OR

'osficar'/exp OR 'osficar' OR 'oslene'/exp OR

'oslene' OR 'osteofar'/exp OR 'osteofar' OR

'osteofos'/exp OR 'osteofos' OR 'osteopor'/exp OR

'osteopor' OR 'osteosan'/exp OR 'osteosan' OR

'osteovan'/exp OR 'osteovan' OR 'osticalcin'/exp

OR 'osticalcin' OR 'porosal'/exp OR 'porosal' OR

'sodium alendronate'/exp OR 'sodium alendronate'

OR 'steovess'/exp OR 'steovess' OR 'teiroc'/exp

OR 'teiroc' OR 'tibolene'/exp OR 'tibolene' OR

'voroste'/exp OR 'voroste' OR 'alendronic

acid'/exp OR 'alendronic acid' OR ((((('1 hydroxy

2':ti,ab,kw AND '3 pyridinyl':ti,ab,kw AND

'ethylidene 1, 1 bisphosphonic acid':ti,ab,kw OR

'1 hydroxy 2':ti,ab,kw) AND '3 pyridyl':ti,ab,kw

AND '1, 1 ethanebisphosphonic acid':ti,ab,kw OR

'1 hydroxy 2':ti,ab,kw) AND '3 pyridyl':ti,ab,kw

AND 'ethylidene 1, 1 bisphosphonate':ti,ab,kw OR

'1 hydroxy 2':ti,ab,kw) AND '3 pyridyl':ti,ab,kw

AND 'ethylidene 1, 1 bisphosphonic acid':ti,ab,kw

OR 2:ti,ab,kw) AND '3 pyridinyl':ti,ab,kw AND '1

hydroxyethylidene 1, 1 bisphosphonic

acid':ti,ab,kw) OR 'acrel':ti,ab,kw OR

'actonel':ti,ab,kw OR 'actonel once a

week':ti,ab,kw OR 'atelvia':ti,ab,kw OR

'benet':ti,ab,kw OR 'ne 58019':ti,ab,kw OR 'ne

58095':ti,ab,kw OR 'ne58019':ti,ab,kw OR

'ne58095':ti,ab,kw OR 'optinate':ti,ab,kw OR

'ribastamin':ti,ab,kw OR 'risedronate':ti,ab,kw

OR 'risedronate monosodium

hemipentahydrate':ti,ab,kw OR 'risedronate

sodium':ti,ab,kw OR 'risedronic acid':ti,ab,kw OR

teriparatide:ti,ab,kw OR 'denosumab':ti,ab,kw

#2. 'randomized controlled trial'/exp

#1. ('decalcification, pathologic'/exp OR

'decalcification, pathologic' OR 'endocrine

osteoporosis'/exp OR 'endocrine osteoporosis' OR

'osteoporotic decalcification'/exp OR

'osteoporotic decalcification' OR 'pathologic

decalcification'/exp OR 'pathologic

decalcification' OR 'osteoporosis'/exp OR

'osteoporosis') AND ('males':ti,ab,kw OR

'man':ti,ab,kw OR 'men':ti,ab,kw OR

'male':ti,a

| Software and Version | Stata 18.0 (StataCorp LLC, College Station, TX) | R 4.3.1 (R Foundation for Statistical Computing, Vienna, Austria) |
| --- | --- | --- |
| Core Functions/Packages | netinstall github("https://github.com/remlapmot/networkmeta")network setup [effect_size] [se], tau2(reml) | library(gemtc)  model <- mtc.model(network, likelihood="normal", link="identity", linearModel="random", n.chain=4) |
| Iteration Settings | network meta i  network meta c | results <- mtc.run(model, n.adapt =50000, n.iter = 100000, thin = 1) |
| Local Inconsistency Test | network sidesplit all, tau | resultnodesplit <-mtc.nodesplit(network, n.adapt = 20000, n.iter= 50000, thin = 1, n.chain=4,likelihood="binom",link="logit",linearModel="random") |
| Heterogeneity Analysis |  | resultanohe <- mtc.anohe(network, n.adapt = 20000, n.iter =50000, thin = 1, n.chain=4,likelihood="binom",link="logit",linearModel="random") |
| Surface Under the Cumulative Ranking(SUCRA) | network rank min, all zero reps(5000) gen(prob) |  |
| Funnel Plot | netfunnel _y _stderr _t1 _t2 , random bycomp add(lfit _stderr _ES_CEN) noalpha |  |

**Supplementary Table 1.** Detailed information about the specific functions or models of STATA and R.

|  | The Global inconsistency | ＞0.05 | DIC of Model of consistency | DIC of Model of inconsistency | The difference is  less than 5 |
| --- | --- | --- | --- | --- | --- |
| Lumbar spine BMD | 0.2957 | yes | 32.19246 | 33.11745 | yes |
| Femoral neck BMD | 0.4667 | yes | 31.06594 | 30.67985 | yes |
| Total hip BMD | 0.7391 | yes | 26.49299 | 27.67321 | yes |
| All adverse events | 0.9508 | yes | 18.98344 | 19.80795 | yes |
| Serious adverse events | 0.6841 | yes | 15.02446 | 14.98956 | yes |

**Supplementary Table 2.** Assessment of model fit. If the difference of DIC value in two modes is within 5, it means that the data is consistent. DIC, deviance information criterion.

**Supplementary Figure 1.** The [forest map](javascript:;) of all outcomes.

a)The results of [forest map](javascript:;) for Lumbar spine BMD.


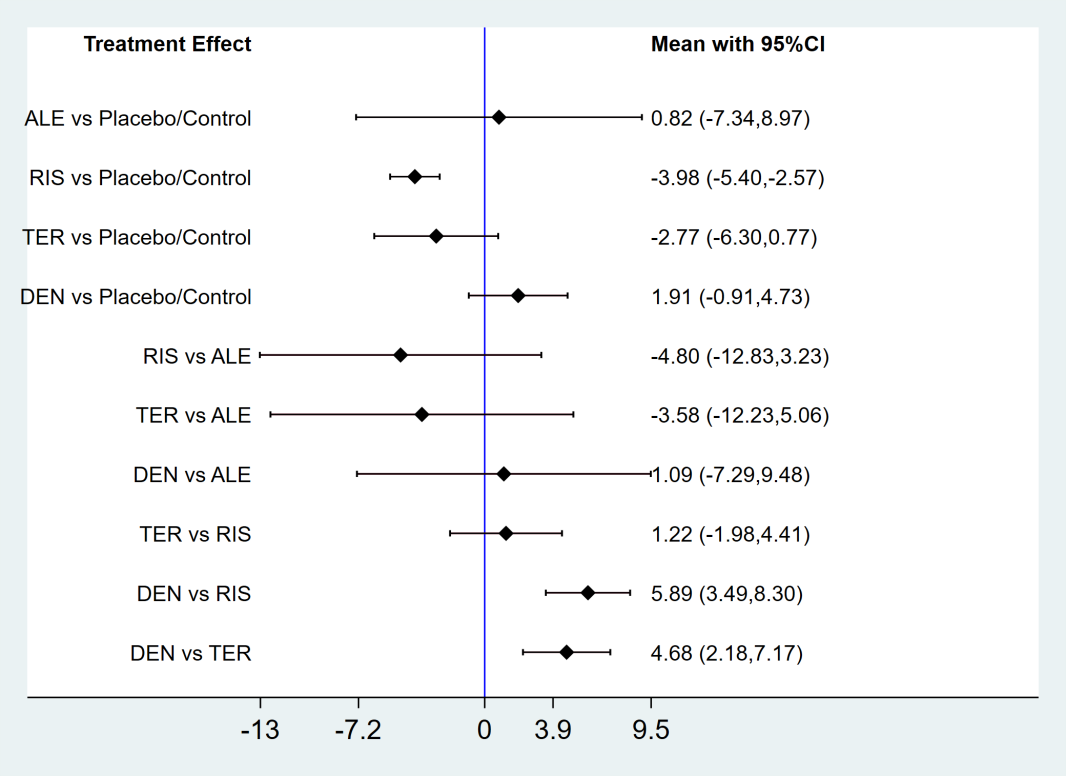


b) The results of [forest map](javascript:;) for Femoral neck BMD.


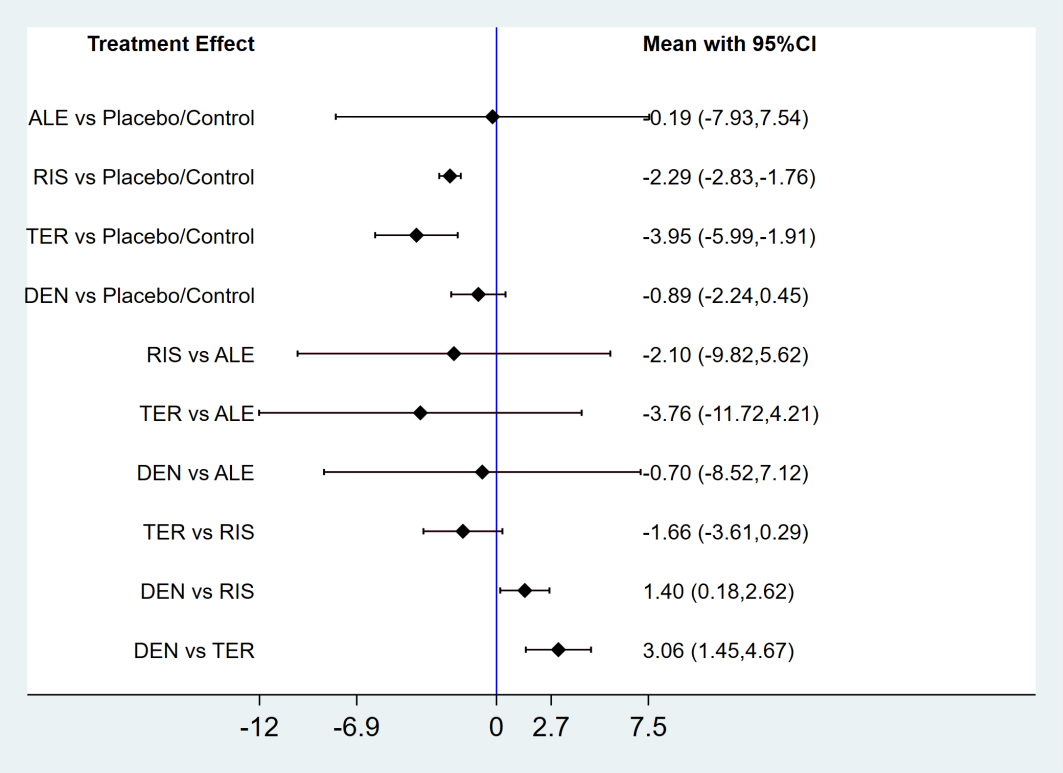


c) The results of [forest map](javascript:;) for Total hip BMD.


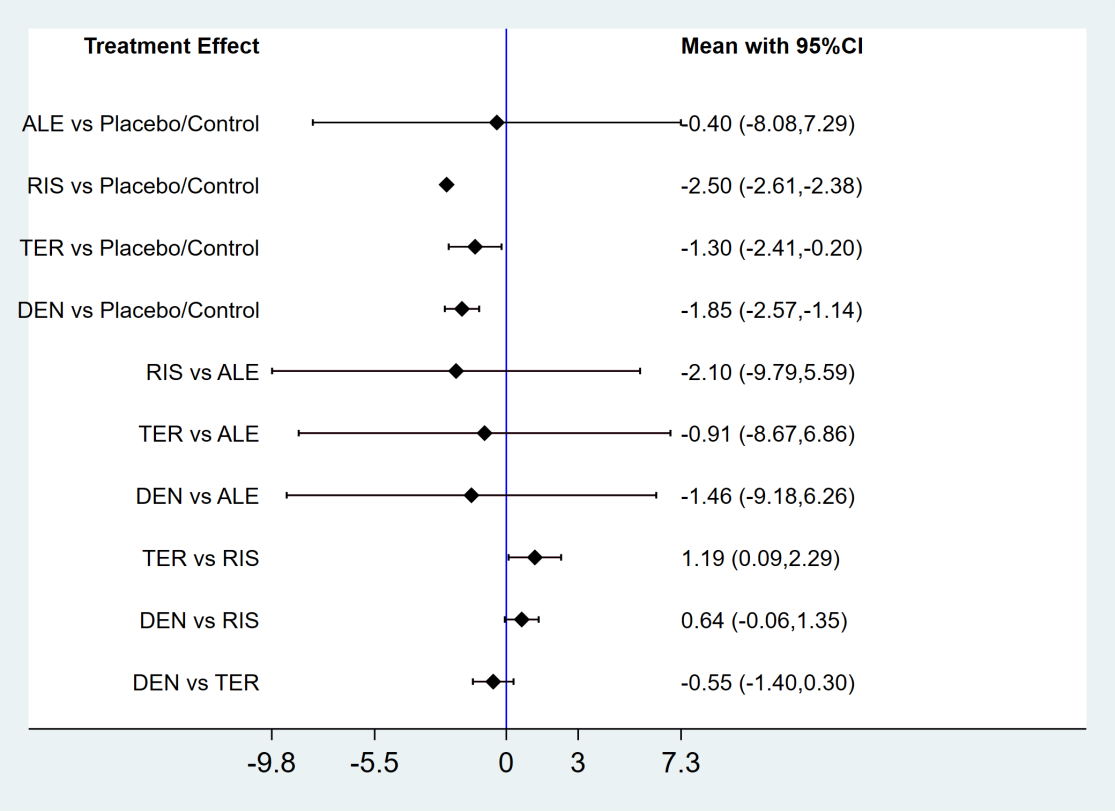


d) The results of [forest map](javascript:;) for All adverse events.


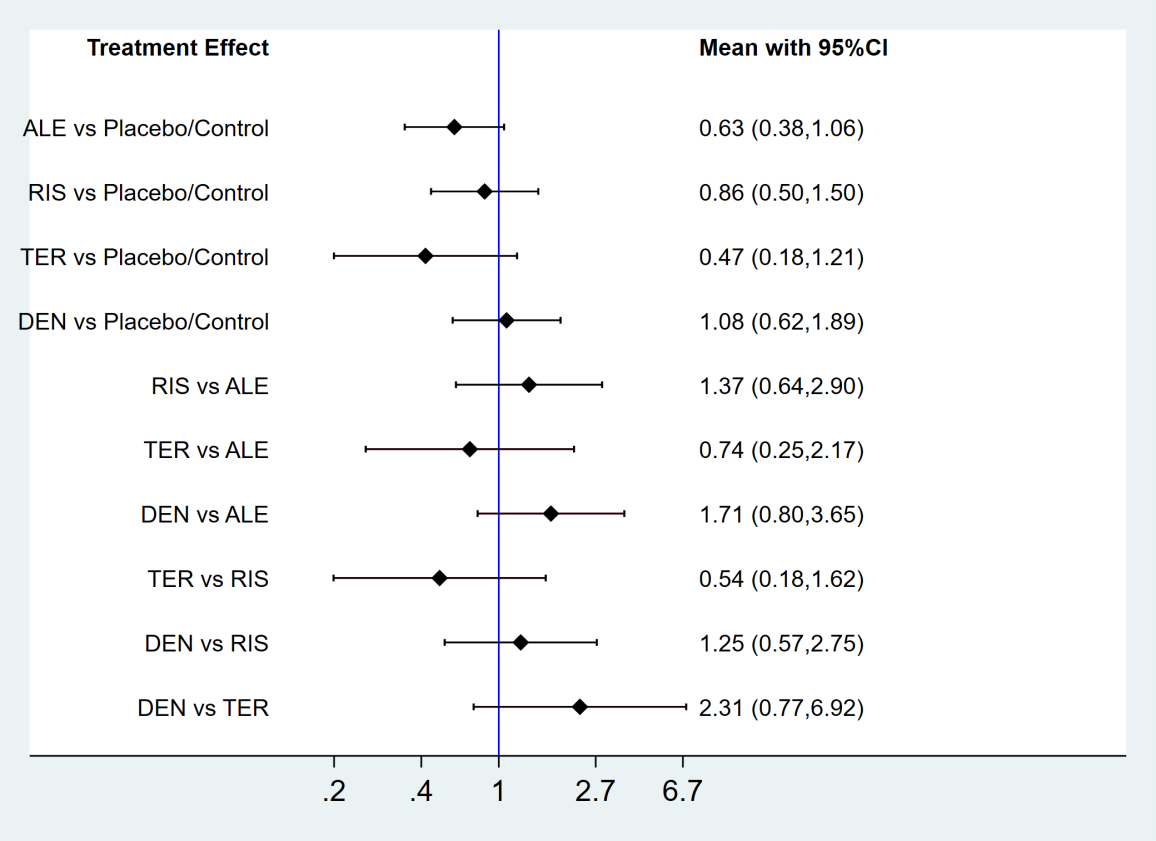


e) The results of [forest map](javascript:;) for Serious adverse events.


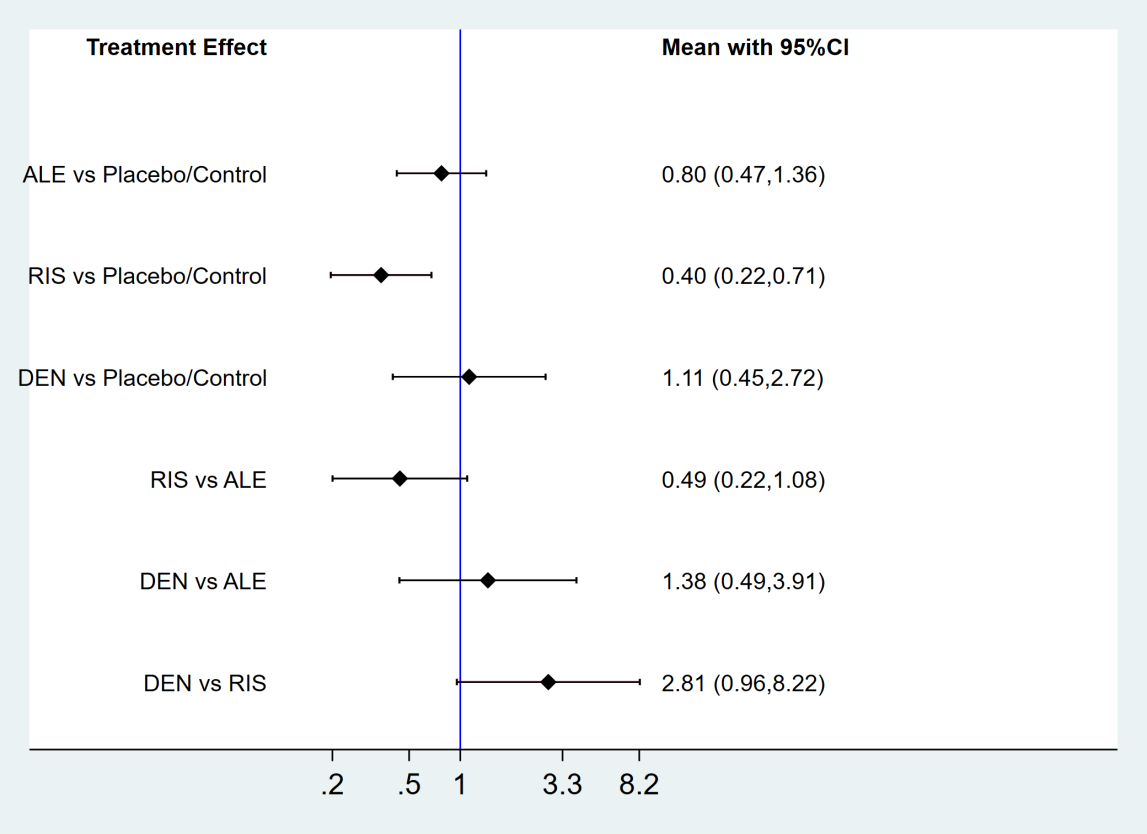


**Supplementary Figure 2.** The network plot of all outcomes.

1. Lumbar spine BMD


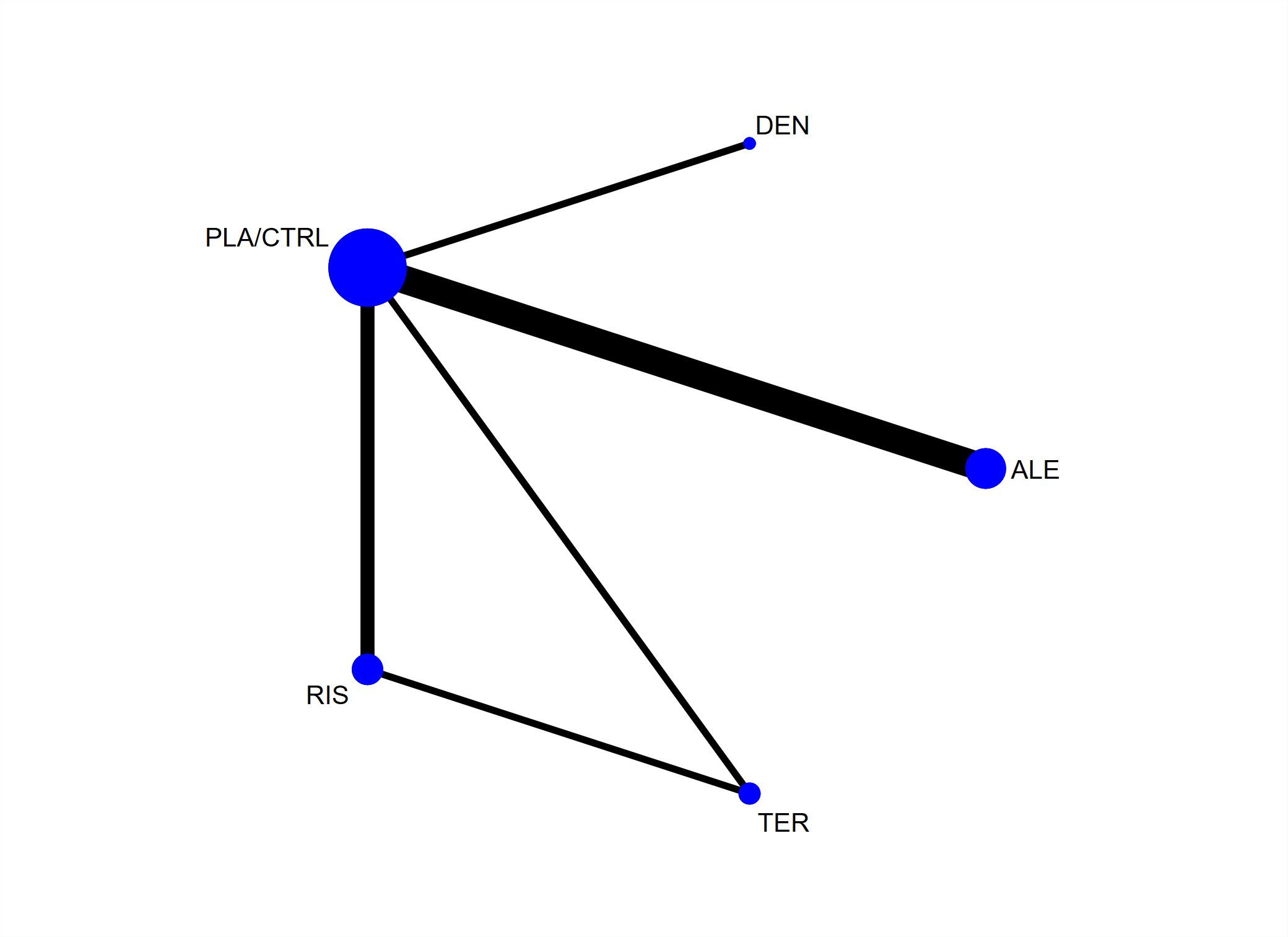


1. Femoral neck BMD


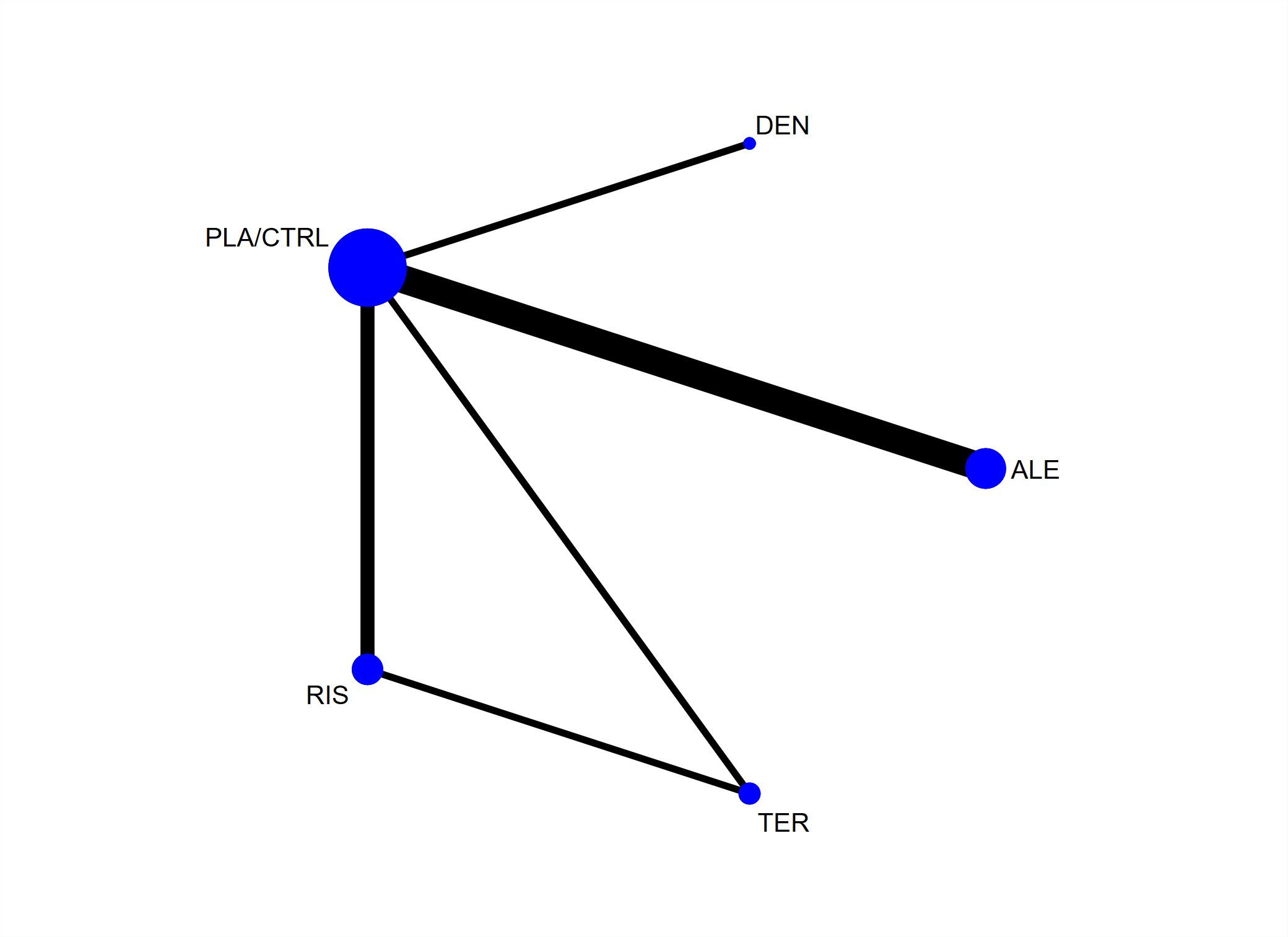


1. Total hip BMD


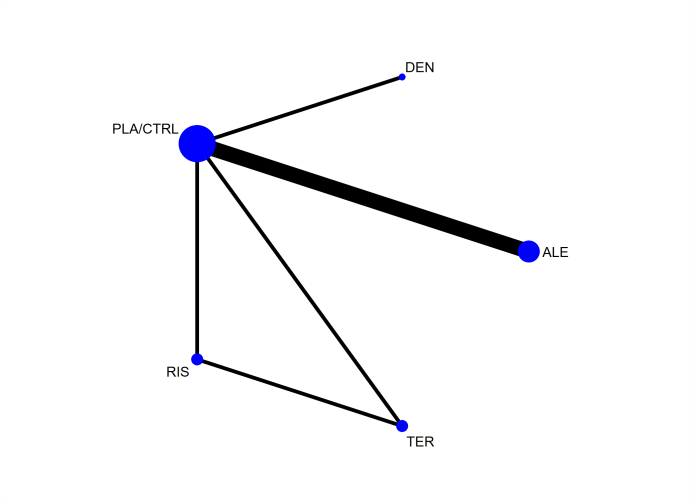


1. All adverse events


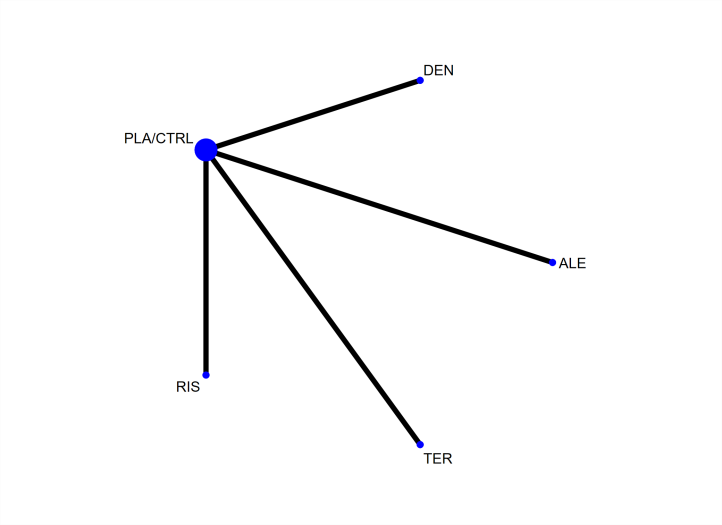


1. Serious adverse events


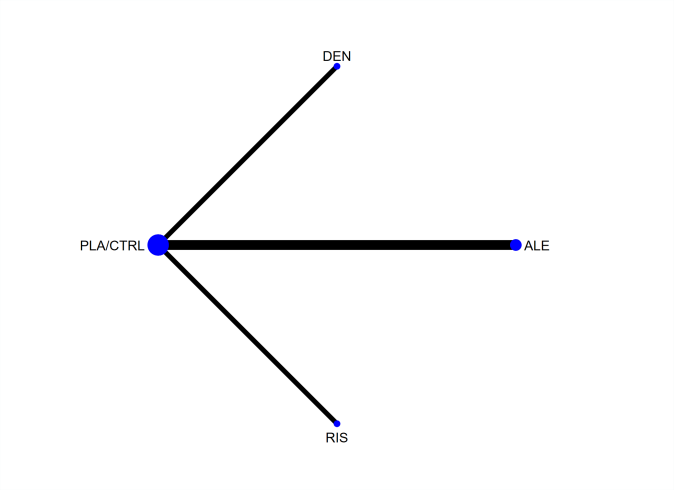


**Supplementary Figure 3.** The Funnel plot of all outcomes.

1. Lumbar spine BMD


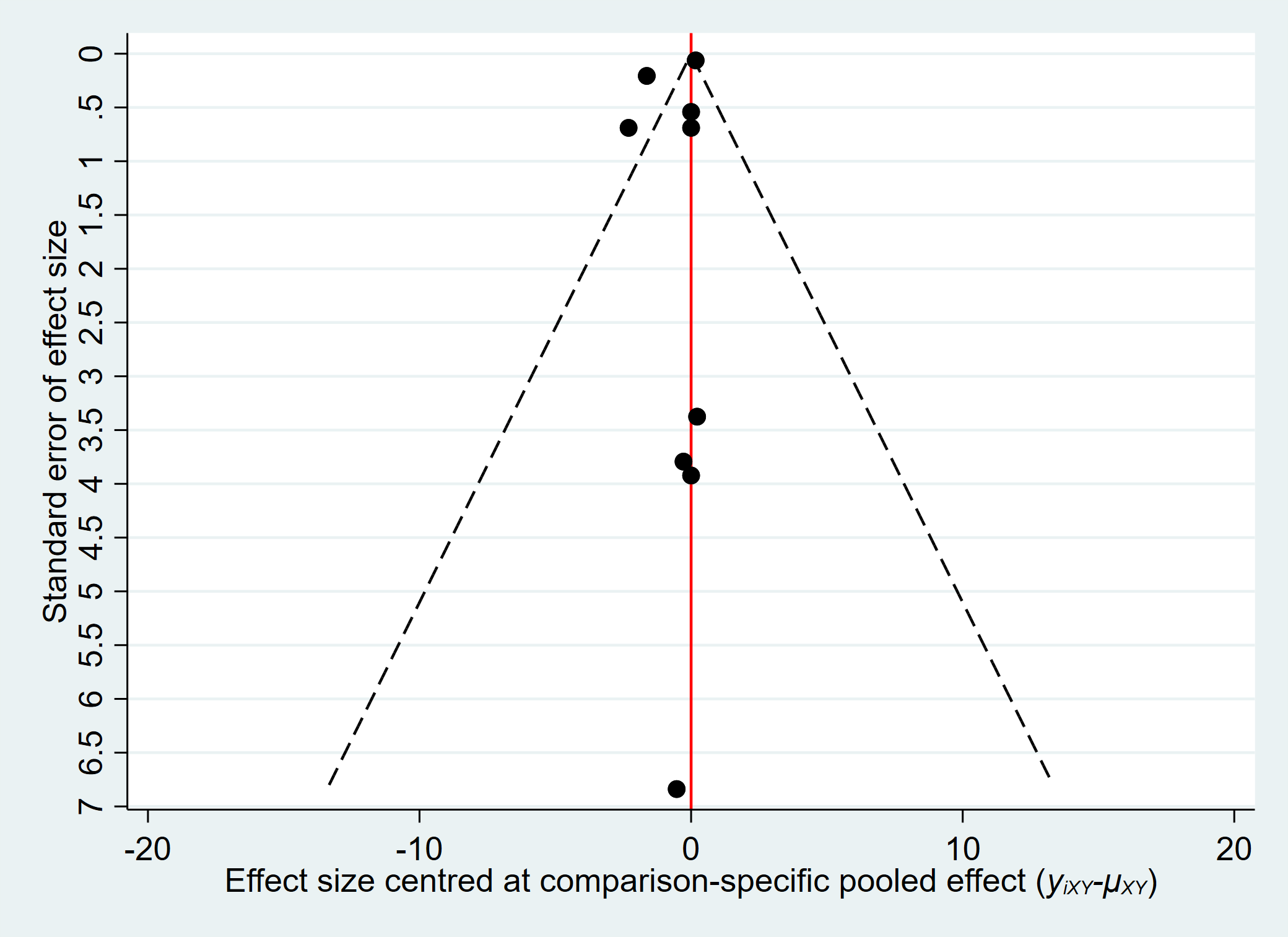


1. Femoral neck BMD


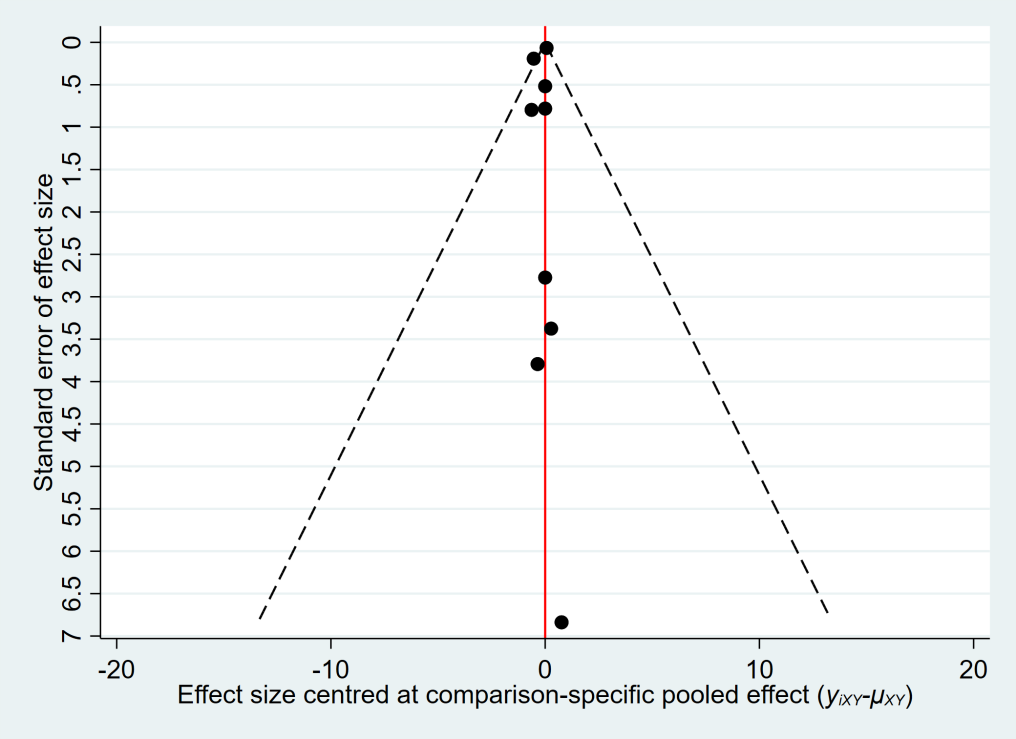


1. Total hip BMD


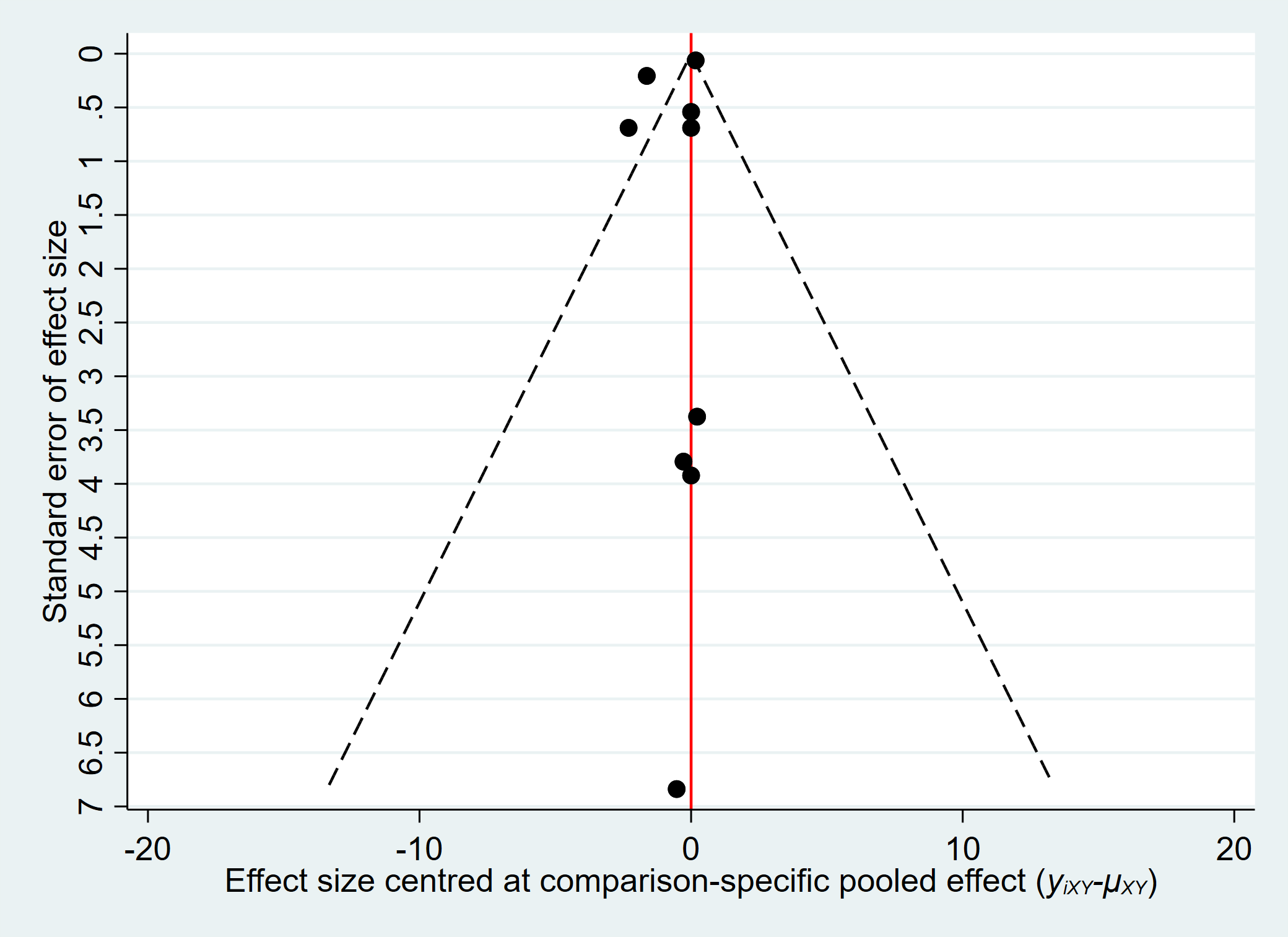


1. All adverse events


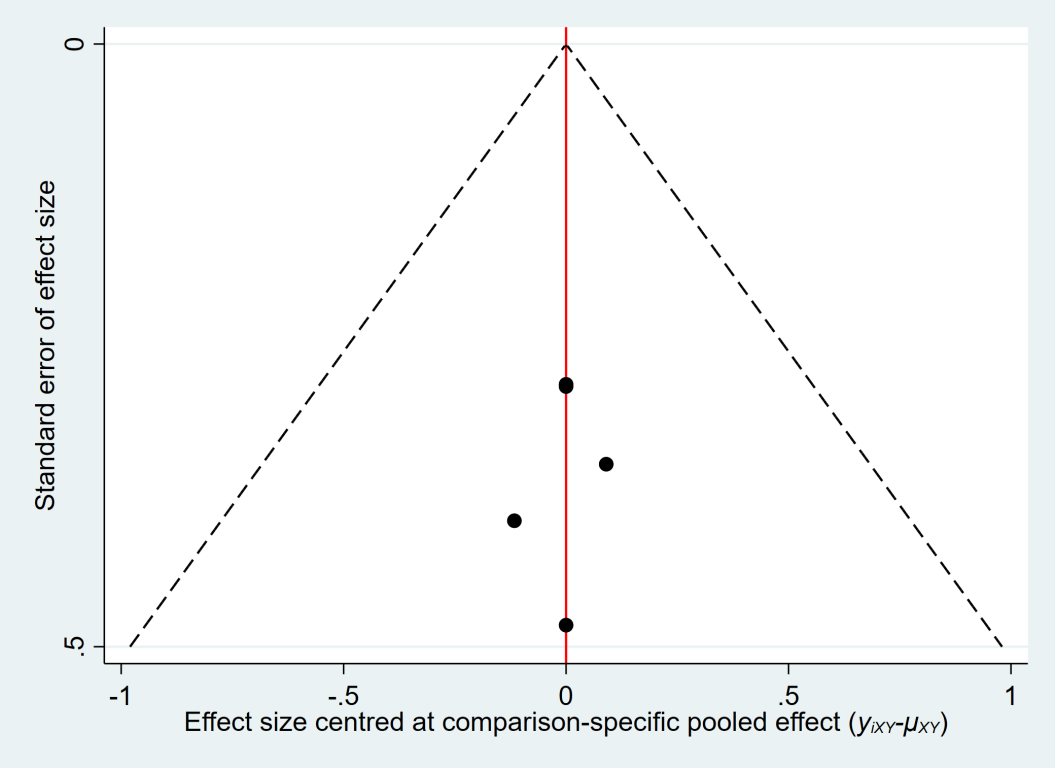


1. Serious adverse events


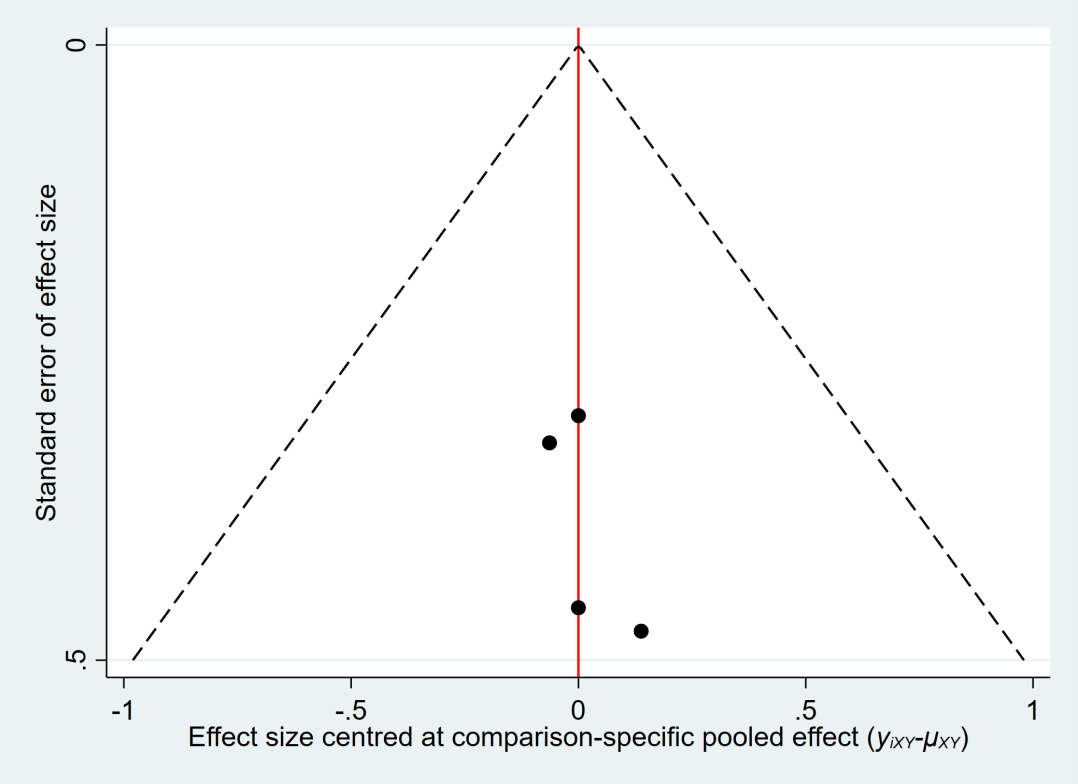

Supplement: Supplementary file 1 [file DataSheet1.docx]
